# Mortality Salience Effects on the Life Expectancy Estimates of Older Adults as a Function of Neuroticism

**DOI:** 10.4061/2010/260123

**Published:** 2010-11-29

**Authors:** Molly Maxfield, Sheldon Solomon, Tom Pyszczynski, Jeff Greenberg

**Affiliations:** ^1^Psychology Department, University of Colorado at Colorado Springs, 1420 Austin Bluffs Pkwy, Colorado Springs, CO 80918-7150, USA; ^2^Psychology Department, Skidmore Collage, 815 N. Broadway, Saratoga Springs, NY 12866, USA; ^3^Psychology Department, University of Arizona, 1503 E. University Boulevard, P.O. Box 210068, Tucson, AZ 85721, USA

## Abstract

Research has shown that reminders of mortality lead people to engage in defenses to minimize the anxiety such thoughts could arouse. In accord with this notion, younger adults reminded of mortality engage in behaviors aimed at denying vulnerability to death. However, little is known about the effects of mortality reminders on older adults. The present study examined the effect of reminders of death on older adults' subjective life expectancy. Mortality reminders did not significantly impact the life expectancy estimates of old-old adults. Reminders of death did however lead to shorter life expectancy estimates among young-old participants low in neuroticism but longer life expectancy estimates among young-old participants high in neuroticism, suggesting that this group was most defensive in response to reminders of death.

## 1. Introduction

Increasing age brings increased awareness that one's remaining lifetime is dwindling. Older adults experience increasingly frequent reminders of mortality due to their own declining health and the deaths of friends and family members. Does this psychological proximity to death increase anxiety and defensiveness among older individuals, or serve as the impetus to develop greater comfort with and acceptance of their mortality? 

 Research examining self-reported fear of death across the lifespan has produced mixed results. Younger adults consistently report higher fear of death than older adults [1, 2], yet it remains unclear whether mortality-related concerns continue to decrease or remain stable in later life. Some studies indicate that fear of death continues to decline throughout later life [3]. However, a meta-analysis of this research concluded that fear of death declines from middle age to old age, but among older adults, age no longer reliably predicts self-reported fear of death [4]. Age differences may depend on the specific aspect of death anxiety being assessed. Cicirelli [5] reported that individuals in mid-old age (75–84) reported greater fears concerning loss of the body (e.g. cremation or bodily decay after death) than those in young-old age (60–74), but the two groups did not differ in reported fears concerning the “unknown” (e.g. what constitutes the afterlife; what it means to cease existing). Research available to date thus provides an incomplete and somewhat puzzling picture of older persons' concerns about death.

### 1.1. Terror Management Theory

Terror management theory (TMT) [6, 7] provides an alternative approach to the study of death anxiety. The theory posits that humankind's capacity for awareness of their inevitable death creates the potential for devastating anxiety that is managed by an anxiety-buffering system consisting of a cultural worldview, self-esteem, and close relationships. The cultural worldview provides a conception of reality and a set of guidelines for valued behavior shared by the culture's inhabitants. Following these guidelines provides structure within an otherwise chaotic world, a sense of belongingness, and literal immortality (e.g. entry into heaven, reincarnation, or other form of afterlife) and/or symbolic immortality (e.g., job promotions, having a park or building named after one's family). Meeting or exceeding culturally constructed standards of value gives the individual self-esteem, the feeling that one is a valuable contributor to a meaningful universe. Close relationships harken back to the security provided by early attachments to one's parents and are essential for the maintenance of both self-esteem and faith in one's worldview. Together, cultural worldview, self-esteem, and close relationships provide a protective shield against the potential for anxiety that results from awareness of the inevitability of death.

Support for TMT comes from a large body of research showing that when people are reminded of death (mortality salience, MS), they show increased commitment to and defense of their worldviews, self-esteem, and close relationships (see [8] for a recent review). For example MS has been shown to lead to more positive reactions toward those who praise one's worldview and more negative reactions toward those who criticize it [9], more self-esteem striving [10], and greater reports of attraction to romantic partners [11]. A recent meta-analysis by Burke and colleagues [12] found that MS effects are highly reliable and yield moderate-to-large effects (*r* = .35, *d* = .75) on a wide range of attitudinal, behavioral, and cognitive dependent variables. 

The pursuit of faith in one's worldview, self-esteem, and close relationships are referred to as distal defenses because they bear no direct or logical connection to the problem of death, but rather, provide protection by enabling people to construe themselves as valued contributors to a meaningful universe. Proximal defenses, on the other hand, refer to defensive responses that deal with the problem of death in a direct and seemingly logical way. Research has shown that reminders of death also increase proximal defenses, such as increasing one's interest in exercise [13] and believing that one possesses characteristics associated with a long life expectancy [14].

### 1.2. TMT and Aging

As people age, it is likely that they become less able to meet many of the standards that previously provided them with self-esteem. This is particularly problematic within Western culture, where many central achievements include success in the areas of career, finances, and physical appearance, all of which are more difficult to accomplish in later life. With advancing age, people are also likely to witness changes in mainstream cultural worldviews, which could result in decreased consensual validation of older adults' cultural worldview, providing fewer opportunities for boosting self-esteem and resulting in drastic changes in the anxiety-buffering system. See McCoy et al. [15] for a theoretical exploration of TMT and aging. 

In contrast to the suggestion of psychological deterioration and struggle in later life, there is abundant empirical support for the idea that older adults developed methods for adapting to the changes inherent in later life. A growing body of research suggests that older adults are increasingly focused on positive information and experiences, while attending less to negative information [16]. As noted above, the literature [1–4] also suggests that older adults generally report lower levels of death anxiety than younger adults, although the details of this pattern are not yet completely clear.

Although many people report that they do not fear death, research suggests that younger adults' self-reported fear of death is not predictive of responses to MS inductions in terror management studies [17]. This raises the possibility that even though older persons report lower fear of death on explicit measures, they may still experience death anxiety and respond defensively to reminders of death. Because TMT posits that individuals with poorly functioning anxiety-buffering systems would be especially susceptible to the influence of mortality reminders, one might expect that older adults would be an especially vulnerable group.

Initial TMT research with older adults indicates that older adults do not respond to reminders of death with the same distal defenses that younger persons use. Specifically, following an MS induction, younger adults were more punitive towards individuals breaking social norms, whereas older participants were more lenient towards moral transgressors [18]. However, this effect only emerged reliably when the reminder of death was very subtle. In other studies, older adults did not show the increased tendency to structure social information in a simplistic manner as displayed by younger adults reminded of death [19] but did show increased generativity striving which was not found in younger adults [20]. 

In studies of proximal defensiveness [21], older participants indicated decreased interest in health promoting behavior when death was in focal attention, in direct contrast to younger and middle-aged adults who showed more interest in health information after death reminders. Conversely, when thoughts of death were no longer in focal attention, older adults with low self-esteem reported increased intention to engage in health behaviors after MS but those with high self-esteem were not influenced by MS. However, it should be noted that older adults in these last two studies ranged in age from 51 to 65 years, providing an incomplete picture of terror management processes in later life. 

The present study assessed the effect of reminders of death on older adults' use of what is perhaps the most direct and simple form of proximal defense—simply believing that death is far away and that one has many remaining years to live. An older adult who has come to accept mortality would be more likely to provide a realistic estimate of his or her lifespan, whereas someone who remains fearful of, or uncomfortable with, death would most likely engage in the proximal defense of denying the event via longer life expectancy estimates. Indeed, it appears that people of all ages are generally accurate in their life expectancy estimates [22, 23]. However, it seems likely that logical awareness of one's likely lifespan would differ from desired lifespan. Consistent with this notion, Cicirelli [5] had participants report the number of years they *thought* they would live and number of years they would *like* to live. Results indicated that mid-old adults (75–84), although probabilistically closer to death, reported a greater discrepancy between the two estimates, suggesting they wanted to live longer than they expected, compared to a young-old (60–74) group asked to make the same estimates. Although this study did not include reminders of mortality, the assessments of both expected and desired life expectancy provide an indication of the contrast existing between real and ideal remaining years in life.

### 1.3. TMT and Neuroticism

Terror management research has shown that some people are better able to deal with death than others. One predictor of responses to reminders of death is neuroticism, a general sensitivity toward fear and anxiety, a trait which may leave highly neurotic individuals less capable of effectively defending against death-related anxiety. Indeed, persons high in neuroticism exhibit exaggerated responses to MS, distancing themselves from the physical body [24, 25] and bodily sensations [26]. Greater defensiveness in response to reminders of death among highly neurotic individuals is perhaps not surprising given research suggesting that neuroticism is associated with greater immediate [27] and longer-term emotional reactivity [28], poorer coping skills [29], increased susceptibility to a variety of psychological disorders [30], and greater death-related anxiety [31]. It is believed that the traits associated with neuroticism leave this group less equipped to manage death-related anxiety. In addition to the role of neuroticism in terror management processes, this personality trait has also been associated with poorer perceived health among older adults [32]; it was therefore hypothesized that neuroticism would be related to older individuals' subjective life expectancy estimates and responses to increased awareness of mortality.

### 1.4. The Present Study

The present study examined effects of death reminders on older adults' subjective life expectancy estimates and the potential moderating roles of age and neuroticism. Bearing in mind Erikson's [33] proposition that the final stages of life are distinctively different from early and midlife, we considered the possibility that old-old participants' (73 to 87 years old) responses to MS would differ compared with young-old participants (57–72 years old). Aside from the intuitive prediction that young-old would expect to live longer than old-old participants, we had no strong expectations about age groups' reactions to MS, but suspected that old-old persons are more accepting of death and would therefore be less likely to respond to MS with longer life expectancy estimates. 

We did however, have clear hypotheses about the moderating role of neuroticism. Based on findings indicating that highly neurotic individuals show exaggerated defensiveness following reminders of morality [24–26], we predicted that participants reporting higher levels of neuroticism would be more likely to respond to MS by distancing themselves from death via longer predicted life expectancies because their anxiety-buffering systems are typically weaker than individuals low in neuroticism, and highly neurotic individuals are more likely to use avoidance-oriented coping mechanisms [34, 35].

## 2. Method

A between-subjects design was used, with two groups: the MS condition and a control condition. Random assignment to condition allowed for distribution of individual difference variables and pre-existing differences in life expectancy beliefs across study conditions. Although having baseline data of participant life expectancy could be useful, it was not assessed because multiple measurement in a short time period could motivate participants to appear consistent or tip them off to the purpose of the study. 

### 2.1. Participants

Participants were recruited from an annual mature adults' minicollege lecture series at a liberal arts college. Attendees expressing interest in participating were given questionnaire packets to complete at home. Of 160 distributed, 134 completed packets were returned (84%). Participants ranged in age from 57 to 87 years old (*M* = 71.44, SD = 6.77). The sample included 26 men and 108 women. Men (*M* = 70.88, SD = 6.69) and women (*M* = 71.57, SD = 6.81) did not differ in age, *P* = .64. (Inclusion of participant sex did not alter analyses reported; there was no main effect for sex, nor did this variable interact with prime, age, and neuroticism, *P*s > .11 for life expectancy estimates, positive affect, negative affect, and self-esteem.) See Table 1 for sample characteristics.

### 2.2. Procedure

To prevent participants from speculating about the purpose of the study, they were told the study concerned the personality and attitudes of older adults. Packets were distributed in sealed envelopes, so experimenters were blind to conditions. Participants were randomly assigned to either the MS (*N* = 73) or pain conditions (*N* = 61); groups did not differ in age (*P* = .46) or gender (*P* = .42).

Participants first completed the 23-item neuroticism subscale of the Eysenck Personality Inventory [36]. Participants indicated agreement with a variety of statements (e.g., Would you call yourself tense or “highly-strung”?) with yes or no. Yes responses were scored as 1, and no responses scored as 0, for a possible range of scores from 0 to 23; higher scores indicate greater neuroticism (*M* = 7.88, SD = 4.92). This measure (*α* = .85) served the dual purpose of assessing the hypothesized moderating variable as well as supporting the cover story. 

Next, participants completed the two open-ended questions for the MS manipulation. MS participants responded to the questions “Please briefly describe the emotions that the thought of your own death arouses in you” and “Jot down, as specifically as you can, what you think will happen to you as you physically die and once you are physically dead.” Control participants responded to parallel questions (“Please briefly describe the emotions that the thought of experiencing intense physical pain arouse in you” and “Jot down, as specifically as you can, what you think will happen to you as you experience intense physical pain”). Physical pain was selected as a control because it is a negative experience that does not involve death. These questions increase the accessibility of, or prime, thoughts related to mortality or the experience of physical pain. Indeed, previous research reveals that MS questions have reliably proven to increase death-thought accessibility as well as defensive responses, whereas questions regarding pain have not [11, 37].

Participants then completed the 60-item Expanded Positive and Negative Affect Schedule (PANAS-X) [38], indicating on a scale of 1 (very slightly or not at all) to 5 (extremely), the extent to which they were experiencing listed emotions (e.g., interested, ashamed) “at the present moment.” Scales of both positive (*α* = .78) and negative affect (*α* = .85) had acceptable reliability. They also completed the 20-item State Self-Esteem Scale [39], indicating on a scale of 1 (not at all) to 5 (extremely) the extent to which they agreed with 20 statements relating to self-esteem (e.g. “I feel that others respect and admire me.”; *α* = .89). Mean scores were calculated for affect and self-esteem, with resulting possible scores ranging from 1 to 5. Although affect and self-esteem are not typically affected by MS in studies with younger adults, we included these measures because little is known about MS effects among older adults. Further, because life expectancy estimation has not been used as a dependent variable in TMT research, assessments of affect and self-esteem allowed us to examine its relationship to these variables. The two measures also provided a delay and distraction before the dependent variable to reduce the likelihood of participants making an explicit connection between the MS induction and dependent measure. 

Participants then reported their gender and age. Lastly, for our primary dependent variable, participants were asked to indicate “To what age do you expect to live?” They then attended a lecture given as a portion of the course they were enrolled in, which included a debriefing and discussion of the study.

## 3. Results

### 3.1. Life Expectancy

The primary dependent variable, remaining life expectancy, was created by subtracting current age from estimated life expectancy. For example, a 65-year-old estimating she will live to 85 would receive a score of 20. Life expectancy served as a dependent variable in hierarchical regression analyses with MS condition, age, neuroticism, and resulting two- and three-way interactions as predictors. Following Aiken and West's [40] methods, the MS variable was dummy-coded, and continuous variables, age and neuroticism were centered at the mean. Main effects were entered in step 1 (Δ*R* ^²^ = .39, *P* < .01), two-way interactions in step 2 (Δ*R* ^2^ = .06, *P* < .01), and the three-way interaction in step 3 (Δ*R* ^2^ = .03, *P* < .01). For the full model, adjusted *R*² = .46, *P* < .01.

Main effects were observed for age, *B* = −.70, SE = .08, *t* = −8.81, *P* < .01 (_partial_ *r* ^²^ = .37), and neuroticism, *B* = −.22, SE = .11, *t* = −1.99, *P* = .05 (_partial_ *r*² = .03); advanced age and higher levels of neuroticism predicted shorter subjective life expectancy. Participants in the MS condition reported marginally lower life expectancy than those in the control condition, *P* = .11.

**Figure 1:**
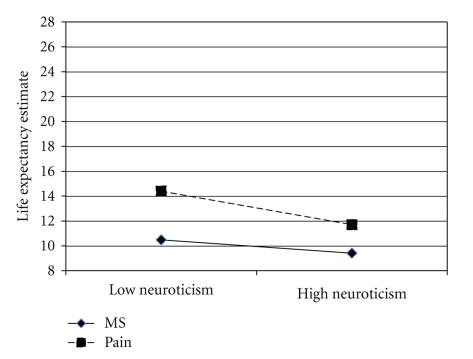
Life expectancy estimates as a function of mortality salience and neuroticism among old-old adults. Note that scores indicate estimated remaining years to live.

These main effects were qualified by a significant MS × neuroticism interaction, *B* = .73, SE = .21, *t* = 3.50, *P* < .01 (_partial_ *r*² = .09), and the predicted significant three-way interaction, *B* = −.08, SE = .03, *t* = −2.90, *P* < .01 (_partial_ *r* ^2^ = .06). To explore this three-way interaction, we assessed the MS × neuroticism interaction separately for young-old (57–72, *M* = 66.38, SD = 3.62) and old-old participants (73–87, *M* = 77.68, SD = 3.91). This analysis revealed no effects among old-old participants, *P*s > .50. Among young-old, there was a tendency for higher neuroticism to predict shorter life expectancy (*P* = .06), and the predicted MS × neuroticism interaction was significant, *B* = .61, SE = .32, *t* = 4.42, *P* < .01 (_partial_ *r* ^2^ = .22). Thus old-old participants' life expectancy estimates were not affected by MS or neuroticism; see Figure 1. 

**Figure 2:**
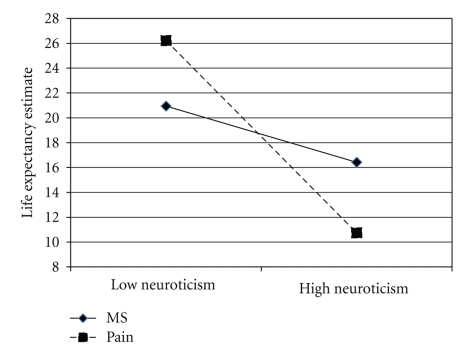
Life expectancy estimates as a function of mortality salience and neuroticism among young-old adults. Note that scores indicate estimated remaining years to live.

Among young-old participants, high neuroticism was associated with lower life expectancies: in the control condition this relationship was statistically significant, *B* = −.94, SE = .18, *t* = −5.15, *P* < .01, but in the MS condition it fell short of significance, *B* = .45, SE = .26, *t* = 1.72, *P* = .09. To test our primary hypotheses about the effect of MS on high and low neurotics, we assessed the effect of MS separately at 1 SD above and 1 SD below the neuroticism mean [34]. Among low neurotic young-old individuals, MS decreased life expectancy estimates, *B* = −5.80, SE = 2.02, *t* = −2.87, *P* < .01. However, consistent with our primary hypothesis, among high neurotic young-old participants, MS leads to longer life expectancy estimates, *B* = 7.93, SE = 2.24, *t* = 3.55, *P* < .01, see Figure 2. The finding that neuroticism was generally associated with reporting lower life expectancies appears consistent with previous findings that neuroticism is associated with poorer perceived health among older adults [32]. However, the finding that high neurotic young-old participants were the only group to respond to MS with increased life expectancy estimates suggests that they are also especially defensive in this regard when thoughts of death are primed.

Looked at differently, young-old participants reported greater life expectancies than old-old participants in all but one condition (*P*s < .01), high neuroticism control (*P* = .15). Reminders of death seemed to counteract this tendency by activating death-denying responses that elevated young-old high neurotics' life expectancy estimates above those of old-old high neurotic participants, *P* < .01, but still not to the level of low neurotic young-old participants, *P* = .09.

### 3.2. Affect and Self-Esteem

One-way analysis of variance was used to test for possible effects of MS on positive and negative affect and self-esteem, and revealed no MS effects on positive affect (*P* = .97) or self-esteem (*P* = .74). Participants in the MS condition tended to report higher negative affect (*P* = .06). Treating positive and negative affect and self-esteem as covariates in regression analyses did not significantly influence the reported results concerning life expectancy; the three way interaction remained significant, *P* = .02. Positive affect was positively related to life expectancy estimates, *B* = .28, SE = .94, *t* = 3.42, *P* < .01, while negative affect was negatively related to these estimates, *B* = −.19, SE = 1.46, *t* = −2.19, *P* < .05.

## 4. Discussion

The present results suggest that the way older adults cope with increased proximity to death depends on both age and neuroticism. Except for those in the high neurotic control group, old-old participants predicted fewer remaining years of life than young-old participants. Young-old participants with lower levels of neuroticism responded to death reminders with lower estimates of life expectancy. Among young-old adults, this arguably more adaptive personality type seems to allow for less defensive responses to reminders of mortality. 

On the other hand, young-old adults who were high in neuroticism responded to MS with longer life expectancy estimates. This is consistent with research linking high levels of neuroticism to the use of avoidance as a defense mechanism [34, 35] and especially defensive responses to reminders of mortality among younger adults [24–26]. Neuroticism predicts both poorer perceived health [32] and greater susceptibility to anxiety disorders [41] among older adults. This is likely reflected in the control condition, in which more neurotic participants generally reported a lower life expectancy. With poorer perceived health and more anxieties, a lower life expectancy may be expected for highly neurotic individuals. For the young-old, this difference between low and high neurotics was eliminated because the high neurotics increased their life expectancy estimate after MS. These findings are consistent with our interpretation of the MS-induced increase in life expectancy among young-old participants in the present study being a proximal terror management defense to avoid the problem of death. As predicted, high neurotic young-old adults increased their life expectancy estimates in the present study, presumably because they lack adequate anxiety-buffering mechanisms. The present results suggest that, generally speaking, high neurotic young-old adults have lower subjective life expectancy than their less neurotic counterparts; however, reminders of mortality activate the defensive reaction of increasing their life expectancy estimates. 

Interestingly, old-old participants did not show this defensive response to MS, regardless of neuroticism level. It is certainly easy to imagine a well-adjusted (i.e., low neurotic) old-old individual accepting the reality of mortality and being less impacted by reminders of it, as found among young-old low neurotic participants in this study. The fact that highly neurotic old-old participants in the present study were also unaffected by reminders of death suggests that with highly advanced age, people may become increasingly accepting of their mortality and thus less likely to engage in defensive denial. It may also be that increasing life expectancy was simply implausible for persons of this age group. The implausibility of denial may play a role in promoting death acceptance.

Taken as a whole, the present results suggest that the process of aging and the associated more frequent reminders of mortality provide motivation for young-old adults low in neuroticism to strive for death acceptance, or at the very least, sober realism, as reflected in their shorter estimates of life expectancy following MS. However, those in the young-old range with higher levels of neuroticism may lack similar psychological resources and therefore attempt to distance themselves from death via longer life expectancy ratings. Yet among old-old individuals, neuroticism was no longer predictive of defensive reactions to MS. This could reflect a relinquishing of efforts to defend, or perhaps with highly advanced age, neurotic individuals develop different, untapped methods for coping with death-related anxiety. Additional research will be needed to determine whether the present results reflect acceptance of death or a switch to other types of defenses.

Erikson [33] suggested that in later life, people contemplate the fundamental nature of their existence. He maintained that in the eighth stage of development, people enter a period characterized by either ego integrity or despair. He described ego integrity as an acceptance of the life led and awareness that death is a natural part of life; these individuals would presumably respond less defensively to reminders of mortality. Others may experience despair, characterized by fearfulness that life is coming to a close and an inability to identify a coherent sense of purpose and/or achievement in their lives. Although a direct measurement of Erikson's concepts of ego integrity and despair was not included in the present study, persons with high levels of neuroticism seem less likely to achieve ego integrity. Erikson's suggestion that despair is characterized by fearfulness, some of which is specific to death [33], and research linking neuroticism to the fear of death [31], are consistent with the possibility that a lack of ego integrity among high neurotics might play a role in the present results. Indeed, young-old participants high in neuroticism displayed defensive distancing from death that might reflect what Erikson identified as a lack of ego integrity. Conversely, people with lower levels of neuroticism predicted shorter life spans following reminders of death, implying the potential acceptance of life's end.

The present findings suggest that with truly advanced age, such as the old-old participants in the present study, neuroticism no longer predicts responses to MS. Joan Erikson's subsequent addition of a ninth stage [42] may help explain this diminishing role of neuroticism toward the end of life. In the proposed ninth stage, which emerges in very old age, individuals must cope with the extreme physical changes experienced in the late 80s and beyond. During this stage, people consider the larger meaning of life, rather than focusing on one's own individual life. Erikson described this as a time involving serious reflection on personal death, which may include acute awareness of the likely causes of one's own passing. It may be that at this point in development, even neurotic individuals move toward greater acceptance of death.

It is important to point out, though, that the old-old group in the present study was largely composed of individuals slightly younger than those expected to be in the ninth stage as defined by Erikson (20 participants were 80 and older, and 4 were 85 and older). It seems likely, though, that the transition toward this ninth stage of death acceptance is a gradual process that begins before the late 80s; indeed, Erikson [33] was clear that the ages specified were general guidelines, that varied across persons, and that the process of moving from stage to stage is a gradual process rather than a discrete transition. The present finding that neuroticism predicts defensive responses to MS in young-old but not old-old adults, suggests that advancing age may encourage changes in coping even among neurotic persons who were resistant to such changes in their younger years.

### 4.1. Limitations and Future Directions

Due to the fact that the study was conducted in a large group as part of an educational program, we were unable to conduct cognitive screens. It would be helpful to be able to link participants' results to their level of cognitive functioning, or at least have definitive information showing that cognitive functioning was not playing a role in the present results. Health information could also be of interest regarding its impact on life expectancy estimates, as subjective health ratings have been shown to predict desired length of life expectancy [22]. We feel confident that the individuals participating in this study were highly functioning and healthy older adults, as all were participating in a lecture series involving transportation to campus and attendance at three lectures a day, one day per week, over the course of 6 weeks. Participants also initiated participation in the study and were actively engaged in the educational program at which they were recruited. Indeed, the high functioning nature of our sample most likely offers a picture of healthy aging processes, which may limit the generalizability of our results to less healthy older adults. Any variability in functioning or health would likely be distributed evenly across conditions due to random assignment. Ideally, we would also have more demographic and personal information, allowing assessment of whether variables such as socioeconomic status, health, and religious beliefs affect responses to MS. However, the homogeneous nature of the participant pool and their overall high level of functioning suggest there were likely to be relatively little variability on these dimensions. Further, in previous TMT studies with older adults, we have not found socioeconomic status, health, or religious beliefs to have significant impact on results [18–20]. 

Another potential limitation may be our control condition. Although research with younger adults indicates that priming thoughts of physical pain produces effects similar to that of neutral primes [11, 37], it may be that thinking about the experience of physical pain is different for older, compared to younger adults, because they are more likely to have health problems that involve physical pain. If their physical pain is associated with a debilitating or potentially fatal disease process, thinking of this pain may subsequently elicit reminders of death. However, a content analysis of participants' responses to the pain salience induction found that only six participants mentioned death-related problems or concepts, and these were no more frequent among old-old neurotic participants than the other groups. (Exclusion of these participants did not significantly alter reported results.) Future studies should include a wider variety of control inductions to identify the most appropriate one for older age groups. 

Despite these limitations, the present results offer insight into older adults' responses to reminders of death and experience of existential anxiety. It appears that older individuals low in neuroticism develop some level of acceptance of their mortality, and as a result they are less likely to respond to MS with significantly longer life expectancy estimates. This may also be the case for old-old individuals regardless of level of neuroticism, whose responses are not significantly affected by MS. However, young-old adults with high neuroticism apparently continue to be motivated to view death as part of the distant future. This suggests that in the early stages of older adulthood, personality variables may be an important determinant of the types of reactions used when reminded of mortality, but that increasingly advanced age promotes less individual differences in these responses.

## Figures and Tables

**Table 1:** Demographic information and individual differences.

	Young-Old	Old-Old	Overall
	*M* (SD)	*M* (SD)	*M* (SD)
Age*	66.38 (3.62)	77.68 (3.91)	71.44 (6.77)
Gender (% Female)	60 (81%)	48 (80%)	108 (81%)
Neuroticism	7.39 (4.67)	8.48 (5.20)	7.88 (4.92)
Self-esteem*	3.92 (.46)	3.64 (.60)	3.80 (.54)
Positive affect	3.28 (.67)	3.18 (.71)	3.23 (.69)
Negative affect*	1.29 (.38)	1.49 (.49)	1.38 (.44)

* indicates age differences ≤.01.
